# Towards Precision Oncology: How Advances in Cancer Genomics, Immunobiology and Artificial Intelligence Will Change Molecular Diagnostics

**DOI:** 10.3390/biomedicines14010175

**Published:** 2026-01-14

**Authors:** Iyare Izevbaye

**Affiliations:** Department of Pathology and Laboratory Medicine, Emory University School of Medicine, Atlanta, GA 30322, USA; iizevba@emory.edu

**Keywords:** precision oncology, hallmarks of cancer, cancer cell biology, immunobiology, artificial intelligence, machine learning, molecular diagnostics

## Abstract

Over the last decades, a significant improvement in cancer patient outcomes has occurred due to advances in cancer cell biology, systemic immunity, tumor-immune microenvironment (TIME) and precision cancer therapy. Despite this explosion of knowledge, its usefulness in clinical practice has been limited by the ability to translate multidimensional data into clinical care. Progress in artificial intelligence (AI) opens up a new frontier, with the promise of achieving synergistic and comprehensive integration. The classification of cancer biology and immunobiology into hallmarks of cancer by Hanahan and Weinberg provides a framework for organizing this information. This systematic classification has enabled the understanding of the interplay and cross-talk between its parts. Targeted cancer therapies and immunotherapies have achieved considerable success, yet their combinatorial potential is still being uncovered. Molecular diagnostics has worked hand-in-hand with precision oncology in deploying new therapies in a cancer-informed and patient-specific way. Harnessing the full power of the advances in these three fields with the aid of AI promises a transformation of molecular diagnostics. This review conceptualizes molecular diagnostics in the context of cancer hallmarks using nonsmall cell lung cancer (NSCLC) as a template, highlighting the potential of a new diagnostic science through the integrative power of AI.

## 1. Introduction

Advances in cancer cell biology, the systemic and tumor-immune microenvironment (TIME) and precision cancer therapy have led to significant progress in patient outcomes. Though they contribute in distinctive ways to outcomes, a highly dynamic cross-talk occurs, modulating and altering their molecular and cellular processes in a pro- or anti-cancer manner.

These advances have warranted a redefinition of cancer. It was traditionally understood as an evolving genetic disease of dysregulated cellular and molecular clonal processes, driven by the progressive accumulation of genetic, genomic and epigenomic transcriptional alterations. Hanahan and Weinberg expanded and conceptualized this definition into a comprehensive framework, which they termed the hallmarks of cancer [1]. The initial cancer hallmarks were predominantly cancer cell-intrinsic or autonomous processes, that is, they described cellular and molecular mechanisms originating within the cancer cell. Since then, the framework has grown to include non-autonomous or extrinsic cancer cell mechanisms, including the tumor microenvironment, the host immune system and the microbiome [2]. Mutations within the genome of the cancer cell hijack normal intrinsic cellular pathways including cell signaling, signal transduction, cell cycle and its regulation, DNA replication, repair and homeostasis, apoptosis and cellular energetics, etc., for its own advantage. The resultant gain-of-function or loss-of-function proffers a growth/survival advantage on the initial cancer cell clone or subsequent subclone, promoting cancer cell dominance.

The Cancer Genome Atlas (TCGA) has comprehensively cataloged mutations, gene expression and epigenomic alterations in many cancer types [3,4]. These alterations can be functionally classified into specific cancer hallmarks [5]. This knowledge has aided drug development that specifically targets functional components of the hallmarks [6]. Designated as targeted therapies, these agents have advanced the principle of personalized medicine, by tailoring therapy to specific alterations in the cancer genome, leading to better efficacy and less toxicity [7].

Molecular diagnostics is well-established in identifying druggable targets and identifying cancer cell-intrinsic hallmarks using advanced techniques (Table 1) [8]. Some categories of cancer hallmarks, e.g., avoiding immune destruction, angiogenesis, microbiome, tumor-promoting inflammation, etc., illustrate the importance of cancer cell-extrinsic mechanisms to cancer evolution and progression [2,9].

The advent of effective immunotherapies in a wide variety of cancer types highlights the growing clinical relevance of these cancer hallmarks and the need to incorporate them into molecular diagnostics and therapeutic prediction [10]. Cancer cell-extrinsic hallmarks have high complexity. Cancer immunobiology displays a complex interactive array of cellular constituents, bioactive molecules and molecular mechanisms. It is cancer-specific, patient-specific and highly dynamic. Elucidating mechanisms and applying them in a patient-specific context within a clinical setting is a goal of personalized medicine [11,12].

**Figure 1:**
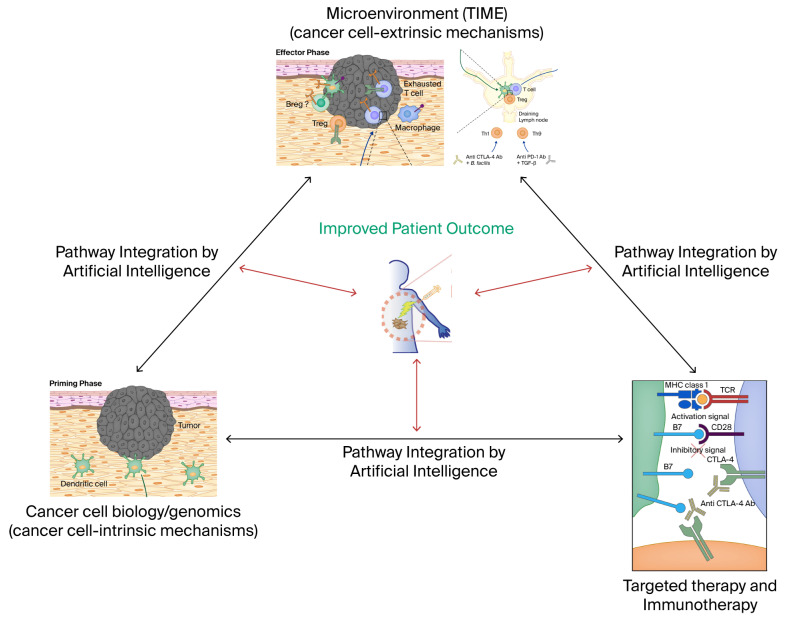
Patient-specific data on molecular pathways of cancer cell biology, systemic and tumor-immune microenvironment (TIME) and cancer therapy can be integrated by artificial intelligence to improve clinical outcomes.

**Figure 2:**
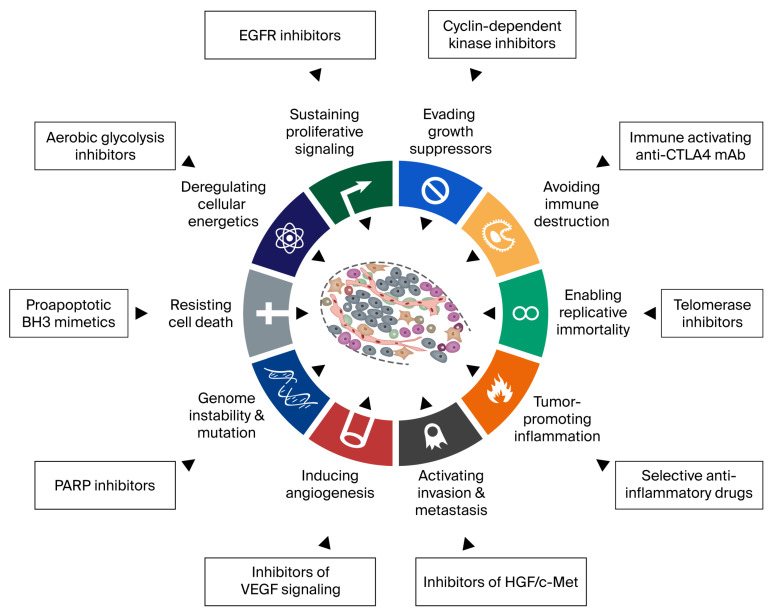
Hallmarks of cancer and the drug inhibitors at each hallmark. Figure reproduced with permission from *Cell* journal [9].

These advances pose a new challenge for personalized diagnostics. This review conceptualizes molecular diagnostics within the framework of the hallmarks of cancer using nonsmall cell lung cancer (NSCLC) as a prototype. It discusses novel approaches to meet these challenges, and the need for a comprehensive, multi-modal synthesis of cancer genomics and immunobiology into precision oncology. It concludes that emerging tools of artificial intelligence (AI) will facilitate the integration of complex data into actionable knowledge. It will also aid in the systematic design of combinatorial therapies targeting cancer vulnerabilities, pathway compensation and escape and resistance mechanisms in each individual cancer (Figure 1 and Figure 2).

This review is divided into three sections. The first parses cancer biology of NSCLC within the framework of cell-intrinsic mechanisms, highlighting mechanistically designed therapies. The second focuses on cell-extrinsic hallmarks, particularly the systemic and tumor microenvironment, cancer-immune cross-talk and emerging biologically based immunotherapeutic strategies. The concluding section summarizes how integrative AI promotes clinical actionability.

## 2. Cell-Intrinsic Molecular Mechanisms of NSCLC and Targeted Therapy

### 2.1. Sustaining Proliferative Signaling

NSCLC cells acquire the property of unrestrained growth from persistent stimulation. This is the cancer hallmark of sustained proliferative signaling. Constitutively activating mutations liberate cancer cells from the controlled and synchronized growth of normal tissue. Growth-promoting signaling pathways that undergo aberrant perturbation in NSCLC include the RAS–RAF–MAPK pathway, PI3K-protein kinase B, Akt and mTOR pathway [5]. These perturbations result from upstream constitutionally activating mutations in genes within these pathways. NSCLC-specific oncogenes include *EGFR*, *ERBB2*, *MYC*, *KRAS*, *BRAF*, *MET*, *CCND1*, *CDK4*, *BCL2* and gene fusions in *ALK*, *NTRK1/2/3*, *ROS1* and *RET*, among others [13]. These driver oncogenes are targets for tyrosine kinase inhibitor (TKI) therapy. Developing or FDA-approved TKIs include 1st, 2nd and 3rd generation EGFR TKI [gefitinib, Erlotinib, Afatinib, Osimertinib]; TKIs against *ALK* and *ROS1* fusion and *MET* amplification (Crizotinib); *RET* (Selpercatinib, Pralsetinib); *KRAS* (adagrasib, Sotorasib); *BRAF* (Vemurafenib); *HER2* (Pyrotinib), etc. [6].

### 2.2. Evading Growth Suppressors

NSCLC utilizes the cancer hallmark of evading growth suppressors as a tumorigenic mechanism through resisting inhibitory signals. NSCLC acquires mutations in tumor suppressor genes (TSGs), including the central gatekeepers, such as *TP53* and *RB1*. In fact, *TP53* is one of the most frequently occurring mutations in NSCLC. Mutations in TSGs confer a growth advantage on NSCLC tumor cells. *TP53* coordinates intracellular signals and modulates multiple processes like cell cycle progression and apoptosis [14]. *RB* transduces extracellular signals and relays these processes to cell growth and proliferative processes like the cell cycle [15]. These two genes act in concert with a network of other genes, resulting in functional redundancy. Other evasive mechanisms in growth suppression include loss of contact inhibition, due to the failure of cell surface adhesion, mediated by genes like *CDH1* (E-cadherin) and *NF2* (Merlin) [16]. The epithelial polarity gene *LKB1* acts to maintain tissue integrity and epithelial structure organization [17]. In NSCLC, mutated genes that function as tumor suppressor genes include *TP53*, *RB1*, *STK11*, *CDKN2A*, *FHIT*, *RASSF1A* and *PTEN* [18]. *TP53* has proven a difficult gene for therapeutic targeting. Potential therapeutic approaches that target this gene include *TP53* gene therapy, the pharmacologic restoration of p53 function, MDM2 inhibition, etc. [19,20,21]. Indirect approaches use *TP53* co-mutation status to inform different therapeutic strategies, including a combination of EGFR-TKIs with chemotherapy, anti-angiogenic drugs or immunotherapy [22].

### 2.3. Resisting Cell Death

Apoptosis and autophagy are major mechanisms in NSCLC for coordinated cell death and anti-tumor activity [23]. Apoptosis is attenuated in cancer cells and plays an important role in the progression to high-grade malignancy and therapy resistance [24]. Genetic alterations that alter the apoptotic machinery include the *BCL-2* gene family, DNA damage sensors including *TP53* via Noxa and Puma proteins, MYC and others [25]. Autophagy is a system for recycling and degrading damaged cellular organelles or cytoplasmic contents [26]. The cancer cells upregulate this process to overcome microenvironmental stress and support proliferation. Signaling pathways that NSCLC cells utilize to recruit the autophagic circuitry include PI3-kinase, AKT and mTOR pathways [27]. The *BCL* family of genes interact with the autophagic pathway through beclin-2. Apatinib is a small molecular anti-angiogenic drug undergoing clinical trials. It triggers autophagic and apoptotic cell death, upregulates cleaved caspase 3, cleaved caspase 9 and Bax and downregulates Bcl-2 in NSCLC cells [24].

### 2.4. Genomic Instability and Mutations

Genomic instability is a characteristic oncogenic mechanism in NSCLC. Impairing genomic integrity and surveillance machinery enhances the multistep accumulation of mutations that confer a competitive advantage on NSCLC subclones. This machinery acts to detect and activate the DNA damage repair processes and cellular mechanisms that intercept or inactivate genotoxic products. Their mechanism of action is similar to caretaker genes, which act like tumor suppressor genes. An important player is the enzyme telomerase, a key gene in the maintenance of the DNA ends called telomeres [28]. Loss of telomeric DNA contributes to karyotypic instability and genomic alterations like amplification and the deletion of chromosomal segments [29]. Copy number changes can be detected by genomic methods, including comparative genomic hybridization and NGS [30]. In NSCLC, these genomic changes include specific allelic loss at 3p, 4p, 9p and 17p. Copy number alterations result in the amplification of *MYC*, *RAS*, *EGFR*, *NKX2-1*, *ERBB2*, *SOX2*, *BCL2*, *FGFR2 AND CRKL*, and the inactivation of *RB1*, *CDKN2A*, *STK11 AND FHIT* [18]. Homologous recombination repair-deficiency (HRD) testing is predictive for PARP inhibitors [31]. It assesses genomic instability by detecting loss of heterozygosity (LOH), telomeric allelic imbalance (TAI), large-scale transitions (LSTs) and mutations in BRCA1/2 genes and other HRD genes [32,33,34]. It is used clinically in ovarian cancers. Clinical trials are evaluating its utility in NSCLC [35].

### 2.5. Activating Invasion and Metastasis

The cancer hallmark of activating invasion and metastasis is a key mechanism in the progression of early-stage curable lung cancer to late-stage disease with distant metastasis. Often called the invasion-metastasis cascade, the serial acquisition of mutations enables cell–cell detachment, enzymatic lysis of the extracellular matrix, then migration and intravasation into nearby blood and lymphatic vessels [36]. After transit in the circulation, extravasation into the parenchyma of distant tissue leads to micrometastasis. Colonization occurs when the cancer cells establish viable, growing lesion. Cancers cells re-engineer an embryonic program in a process called Epithelial to Mesenchymal Transition (EMT) to initiate and facilitate this process [37]. At emergence in distant tissue and with colonization, the cancer cells revert the processes via Mesenchymal to Epithelial Transition (MET) [38]. In lung cancer, pathways and genes associated with the EMT include COX-2, LKB1, WNT, NOTCH, TGFβ, PI3K–Akt and JAK–STAT pathways.

### 2.6. Inducing/Accessing Angiogenesis

NSCLC utilizes the induction of angiogenesis in its growth and progression. Unlike normal adult tissue, in which transient angiogenesis occurs only as part of the wound healing process or in female reproductive cycling, cancer cells permanently activate an angiogenic switch, leading to neovascularization that sustains the expanding tumor [39]. Inducers of this process include well-characterized proteins like vascular endothelial growth factor-A (VEGFR) and proangiogenic signals, e.g., fibroblast growth factors (FGF). Other factors that promote angiogenesis in lung cancer include EGF, FGF-2 and HIF [40].

### 2.7. Enabling Replicative Immortality

Normal cells have limited growth and division cycles, after which senescence or cell death occurs. However, in NSCLC, cells escape this restriction and acquire replicative immortality. The enabling of replicative immortality in NSCLC is a hallmark of cancer. This mechanism occurs through the activation of the enzyme, telomerase. Telomerase typically functions to maintain telomere length. Alterations in its function promote cancer cell immortality and enhance tumorigenesis in NSCLC [41]. Implicated mechanisms include the accumulation of mutations in the context of impaired caretaker gene functions, and other non-telomeric functions, e.g., DNA damage repair, RNA-dependent RNA polymerase, promoting cell proliferation and resistance to apoptosis. High telomerase activity is associated with advanced diseases in NSCLC. Strategies targeting telomerase include antisense oligonucleotide against human telomerase RNA and immunotherapy [42,43]. Explanatory models for replicative immortality include genetic diversity from clonal evolution, cancer stem cell adaptability and cancer cell plasticity [44].

### 2.8. Deregulating Cellular Energetics

The unregulated and excessive growth of NSCLC cells alters energy metabolism in response to increased demand for fuel and nutrients [45]. This hallmark of cancer, deregulating cellular energetics, results in a metabolic reprogramming that switches energy metabolism to predominantly glycolysis, even in the presence of oxygen. Aerobic glycolysis is facilitated by the upregulation of the glucose transporter, GLUT1, and the glycolytic pathway. NSCLC oncogenes, e.g., *RAS*, *MYC* and *TP53*, play a mediatory role. Gain-of-function mutations in *IDH 1/2* are implicated in cancer cell energy metabolism [46]. Evidence suggests they arise by clonal selection for their biochemical property to alter energy metabolism.

### 2.9. Nonmutational Epigenetic Reprogramming

The altered epigenome of NSCLC cells facilitates tumor heterogeneity, unrestrained self-renewal and multi-lineage differentiation [47]. These features are related to the stem-ness in cancer cells and present a major challenge to therapy through the development of chemoresistance. Epigenetic reprogramming occurs through mechanisms including DNA methylation, histone modification by methylation and acetylation and the action of non-coding RNA including microRNA, pi-RNAs, circRNAs and other sncRNAs. DNA methylation is mediated by genes such as the DNMT family of methyl transferases, UHRF1 and TET [48]. MicroRNAs implicated in lung cancer include tumor inhibitory forms, e.g., *let-7* miRNA that regulates *NRAS*, *KRAS*, *MYC* and *HMGA2*. Others include *miR-29a/b/c*, *miR-34-a/b/c* and *miR-16*, etc. [49]. Oncogenic miRNAs enhance cancer development by promoting cell proliferation and antagonizing apoptosis. Examples include the miR-17-92 cluster, which targets *PTEN*, *E2F1-3*, *BIM*, *miR-21*, *miR-93*, etc. [50]. MicroRNAs are potential prognostic and therapeutic biomarkers in lung cancer [49]. In NSCLC, methylation patterns differentiate smokers from nonsmokers. In smokers, high promoter methylation of p16, *MGMT*, *RASSF1*, *MTHFR* and *FHIT* occurs at high frequency, while methylation profiles in *RASSF2*, *TNFRSF10C*, *BHLHB5* and *BOLL* are more commonly observed in nonsmokers [18]. Since epigenetic modifications are reversible and can be altered by pharmacological agents, they present a potential therapeutic target. FDA-approved epigenetic drugs are used predominantly in hematologic malignancies, e.g., Azacitidine (MDS), Decitabine (MDS), Romidepsin (Cutaneous T cell lymphoma), etc. DNMT inhibitors and HDACs are being explored in lung cancer [51]. DNA methylation detection may serve for the detection of early-stage NSCLC in tissue and plasma [52].

### 2.10. Senescent Cells

In NSCLC, loss-of-function TP53 mutations play a role in oncogene-induced senescence, thereby promoting tumorigenesis [53]. Senescence is a physiologic response to cellular stress characterized by stable cell cycle arrest and the release of damage signals of pro-inflammatory factors, e.g., chemokines, cytokines, growth factors and proteases [54]. While acute senescence is considered anti-tumoral, chronic senescence-associated secretory phenotype (SASP) promotes many hallmarks of cancer and facilitates a microenvironment favorable to tumor development. *TP53* induces cell senescence via p21. Advances in the understanding of this process will enable treatment strategies that combine pro-senescence treatments with senolytic or senomorphic agents, e.g., the HDAC inhibitor Panobinostat, which has been shown to possess senolytic effects in senescent cancer cells [55].

### 2.11. Unlocking Phenotypic Plasticity

Tumor cell plasticity is a cancer hallmark seen in NSCLC. The cytomorphologic features of NSCLC, e.g., adenocarcinoma vs. squamous cell carcinoma (SCC), its state of differentiation, e.g., well-differentiated to poorly differentiated, is a result of plasticity. Phenotypic plasticity is an adaptive response to environmental changes that cancer cells acquire and deploy to maintain a fitness advantage by modifying their phenotypic traits [56]. These phenotypic adaptations, including metastatic competency, immune evasion and treatment resistance, mitigate anti-cancer processes and arise throughout the course of cancer development and evolution. Genetic and non-genetic mechanisms, such as epigenetic modifications of the genome, underlie mechanisms of phenotypic switching. Cancer cells may undergo transdifferentiation, blocked differentiation or dedifferentiation in response to environmental cues. In the process, the cancer cells take on a phenotypic state conducive to growth and survival [57]. The molecular and cellular mechanisms of cancer phenotypic plasticity are complex and diverse. Some are repurposed processes in normal development and wound healing.

Understanding the underlying mechanisms will uncover potential targets against shape-shifting adaptive processes that underpin the inevitability of cancer progression [44]. Transdifferentiation in NSCLC may occur through the dysregulation of pathways that promote or suppress plasticity. Upregulated pathways during transdifferentiation include the cell cycle/DNA damage repair pathways, genes in the PRC2 complex, the AKT pathway and the Wnt pathways. Downregulation pathways include anti-tumor immune response pathways [58]. Dedifferentiation may be mediated by HIF1 alpha and HIF2 alpha via SOX2 and Oct4 [59]. Studies in NSCLC show that the epigenetic switch between SOX2 and SOX9 is a potential regulator of cancer plasticity and progression [60]. The amplification of SOX2 is also implicated in the SCC phenotype [61].

## 3. Cell-Extrinsic Mechanisms and Immunotherapy

The TIME is an ecosystem of diverse cells, engineered by the interplay of the growing cancer cell with the stromal and immune system [62]. Three important cancer hallmarks that arise from the TIME include tumor-promoting inflammation, the polymorphic microbiome and immune evasion.

### 3.1. Tumor-Promoting Inflammation

The innate immune cells consisting of cells such as neutrophils, eosinophils, macrophages, mast cells and myeloid-derived suppressor cells exhibit both tumor-promoting and anti-tumor effects through the production of bioactive molecules including growth factors, survival and angiogenic factors and extracellular matrix proteases that aid invasion and metastasis and facilitate other cancer hallmarks. Macrophages are important tumor-associated inflammatory cells. Traditionally categorized as M1 (classical) and M2 (tumor-promoting), macrophage classification has evolved to account for the macrophage phenotypic diversity, resulting from differing ontogeny and local stimuli [63]. They promote survival, development and tumor dissemination via processes like angiogenesis, EMT and immunosuppression [64]. Factors such as interleukin-1 (IL-1) and TNF-alpha lead to the activation of the NF-kB and STAT3 pathways, which induce tumor-forming mutations and produce a self-sustaining cycle that maintains the tumor inflammatory microenvironment. Inflammatory factors associated with lung cancer include IL-1Beta, IL-4, IL-6, IL-11, IL-12, TNF-alpha, MCP-1 and TGF-Beta.

### 3.2. Polymorphic Microbiome

The polymorphic microbiome is a diverse community of resident microorganisms on barrier tissue, particularly the gastrointestinal system, lung, breast and urogenital system. They have an important role in cancer development and progression. Their effects may promote or impede the acquisition of cancer hallmark characteristics. Oral taxa, e.g., Streptococcus, Veillonella, etc. dominate the lower airways of NSCLC patients [65]. Tumor promotion can occur through mutagenesis by genotoxic bacterial toxins and other biomolecules. They may act directly, through DNA damage or by disrupting genome repair mechanisms. An example is the E. Coli PKS locus, which is mutagenic to the human genome [66]. They may also mimic receptor agonists that stimulate epithelial proliferation through pathways such as ERK, PI3K and MAPK [65]. The microbiota interact with various cellular and physiologic processes, including the adaptive and innate immune system, cellular energetic and metabolism, histone modification and cell cycle progression, modulating their cancer activity [67]. In NSCLC, the composition of intestinal flora may be a predictive factor for immunotherapy selection [68].

### 3.3. Avoiding Immune Destruction

The understanding of the immune evasion in NSCLC, a critically important cancer hallmark, has led to advances in therapeutic modalities. Oncogenic mutations occur at a constant rate in normal human cells despite cellular mechanisms to prevent or correct errors during DNA replication. The three processes whereby the systemic immunity interacts with cancer development have been extensively studied [69]. These are immune surveillance, immunoselection and immunosubversion.

In immunosurveillance, the systemic immunity detects and destroys cancer precursors before they become clinically apparent. Immunoselection refers to the emergence of non-immunogenic tumor cell variants as a result of cytotoxic selective pressure. Immunosubversion is the active suppression or hijacking of systemic immunity for the cancer cell’s growth advantage. Failure in immunosurveillance disrupts the equilibrium. Immunoselection then favors an advantaged clone. Eventually, the escape and hijacking of the immune system leads to overt cancer.

Immune cross-talk between the cancer cell and systemic immunity and the TIME occurs in a specific and highly choreographed manner [70]. Cell-intrinsic mechanisms also influence cell-extrinsic mechanisms, potentiating their cancer-promoting effect. For instance, aberrant JAK–STAT signaling leads to uncontrolled proliferation. It also acts on the immune system via interleukin 6 to inhibit the inflammatory response [71].

The site-specific phases of the activation and response of cellular immunity by T cells is called the immune cycle [72]. In the lymphatics, tumor antigens are processed and presented on cell surface MHC class I molecules by antigen-presenting cells (APCs). Binding to T cell receptors (TCRs) leads to T cell priming and activation, facilitated by costimulatory molecules, such as B7. Activated T cells are trafficked back into the TIME, where antigen recognition and tumor cell killing occurs. A cast of proteins, including immune checkpoint molecules, PD-1 and CTLA-4, play a critical role in this process.

PD-1 (programmed death -1) is a cell surface receptor that binds to its ligand PDL-1, [73]. The binding of PD-1 to PDL-1 impairs TCR signaling and T cell activation. PD-1 is expressed on T cells, B cells, myeloid and NK cells. PD-L-1 is expressed on hematopoietic, antigen-presenting cells and non-hematopoietic cells [74].

CTLA-4 (cytotoxic T-lymphocyte associated protein 4) is a costimulatory protein receptor for the TCR and is expressed on regulatory T cells and activated T cells. It binds competitively with CD28 to the ligands B7.1 and B7.2 and suppresses T cell response.

**Figure 3:**
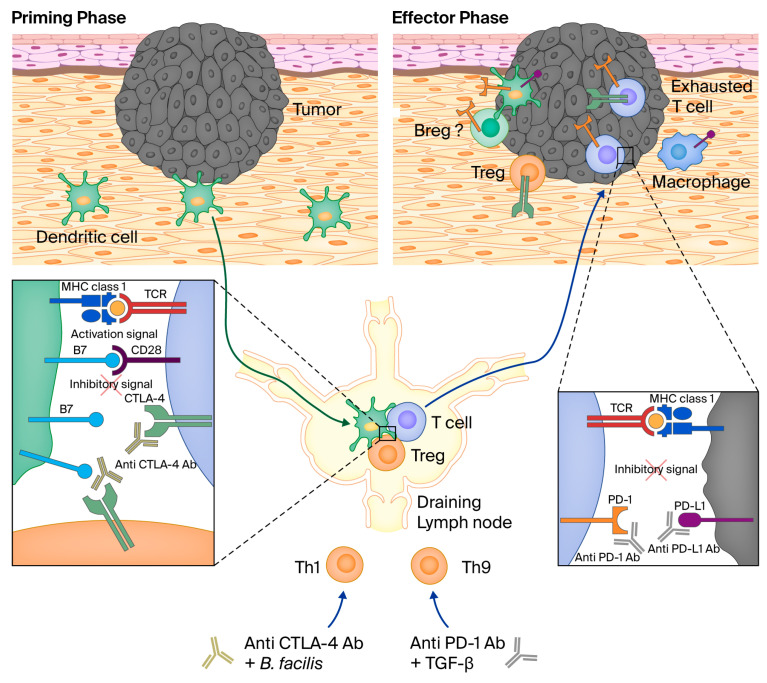
Activity of PDL-1 and CTLA-4-4. A priming phase occurs at initial contact with antigen-presenting cells at tumor site. Activation of cytotoxic and regulation occurs at peripheral lymph nodes with T cells, Th1 and Th9 helper cells. Effector phase involves tumolysis with other immunomodulating and regulating cells. Exhausted T cells result from the downregulation of this process. Anti-CTLA-4 and anti-PD-1/anti-PDL-2 inhibit this process. The activity of CTLA-4 is at the central lymph nodes. Activity of PDL-1 is peripheral at the tumor site. Dotted lines indicate amplification of the area within the box. Figure 1 and Figure 3 reproduced with permission from Frontiers of Oncology under the Creative Commons License [76].

PD-1/PD-L1 and CTLA-4 facilitate the central and peripheral tolerance of systemic immunity (Figure 3) [75]. They promote the negative selection of self-recognizing lymphocytes in primary and secondary lymphoid organs. They also act in the process of immune exhaustion, which is progressive effector T cell impairment due to persistent antigen encounter. This response mechanism occurs to mitigate against tissue destruction from chronic infections. Immune exhaustion is hijacked to downregulate the anti-cancer response as an immune evasion mechanism.

CTLA-4 function occurs at the initial priming stage of naïve T cell activation, primarily at lymph nodes. It binds mainly to costimulatory molecules on professional APCs, e.g., B7, leading to the sequestration and diminution of its TCR stimulatory function and other immune inhibitory roles through Treg cells. PD-1 regulates previously activated T cells at a later effector phase in the cycle within peripheral tissue. PD-1 binds to several ligands, expressed by immune and non-immune cells, including PDL-1/PDL-2, leading to reduced T cell activation.

PD-L1 is expressed in 20–30% of NSCLC, 24–49% of melanoma, 70% of epithelial ovarian cancers and 20% of triple negative breast cancer, at various rates in gastrointestinal malignancies, including 5% of colorectal cancer, 11–30% of cholangiocarcinoma, as well as head and neck, urothelial carcinoma, etc.

### 3.4. Immunotherapy in Cancer

The increasing menu of immunotherapies include immune checkpoint inhibitors (ICIs), therapeutic cancer vaccines (TCVs), bi-specific T cell engagers (BiTEs) and adoptive cell therapies (ACTs) [77].

ICIs block the interaction of inhibitory surface receptors of T cells (PD-1/PDL-1, CTLA-4) and their cognate ligands, leading to an increase in T cell activation and proliferation and enhancing anti-tumor activity. FDA-approved ICIs include anti-CTLA-4 inhibitors (Ipilumumab and Tremelimumab) and anti-PD-1 (Pembrolizumab, Nivolumab) and anti-PDL-1 drugs (Durvalumab and Atezolimab).

TCVs deliver tumor antigens to the systemic immunity and elicit an anti-tumor endogenous T cell response. The antigens may be broadly shared onco-antigens, e.g., KRAS, TP53 or patient-specific neoantigens [77].

BiTEs are recombinant proteins consisting of two distinct single-chain variable fragments linked by a short sequence. These alternatively bind a T cell-activating molecular, e.g., CD3, or a tumor-associated antigen. Synchronous binding of the pair to their targets induces T cell activation and tumor cell destruction.

Adoptive cell therapies (ACTs) involve the infusion of genetically engineered or tumor-infiltrating T cells, after ex vivo expansion, as therapy against tumor cells. Genetic engineering produces a transgenic TCR or a chimeric antigen receptor (CAR) which, on tumor antigen binding, elicits a cytotoxic effect. CAR-T cells are target-versatile and MCH-independent. Factors such as tumor heterogeneity, low lymphocyte penetration and the immunosuppressive tumor microenvironment limit their effectiveness in solid tumors [11].

ADCs are monoclonal antibodies chemically linked to a cytotoxic drug. ADCs combine the specificity of tumor antigen-targeting antibodies with the toxicity of chemotherapy. ADCs effect cytolysis through immunogenic cell death, antibody-dependent cell-mediated cytotoxicity and dendritic cell activation [78].

Cytokine therapy has potential therapeutic roles with other immunotherapies by potentiating antibody-dependent cellular cytotoxicity [ADCC] through the immunomodulatory effect [79].

Oncolytic viruses facilitate tumor lysis by selectively replicating within tumors and activating cytolytic and immune mechanisms for tumor destruction [80]. Only Talimogene laherparepvec, for advance melanoma, has received FDA approval [81].

Immunogenic cell death (ICD) is a newly defined mechanism of tumor cell killing by radiotherapy and chemotherapy. It is characterized by extensive cell lysis with a release of intra-tumor cell fractions, enhanced activation, uptake and presentation of tumor antigens by dendritic cells, increased cross-priming and proliferation of tumor-specific cytotoxic T cells and the production of tumor-specific antibodies [78]. Eliciting the immunogenic response may transform an immunologically cold tumor, otherwise insensitive to immunotherapy, into a hot and immunotherapy-sensitive cancer [82]. ICD inducers include immunomodulatory agents such as TNFRSF9, CD40, immunostimulatory cytokines, cancer vaccines, etc. [82,83,84].

### 3.5. Immune Subtypes in Cancer

The constitutional genetic variability in the human immune system is a significant determinant of the composition of the TIME and its tumor-immune response. Immunogenomics utilizes multiple approaches including NGS, flow cytometry, immunohistochemistry (IHC), single cell sequencing, spatial transcriptomics, bioinformatics and AI [85]. The identification of cancer-immune cross-talk in different classes of immune subtypes may serve as potential targets of immunotherapy. Computational neoantigen prediction can guide adoptive cell therapy and cancer vaccines [85]. A comprehensive TCGA analysis of 10,000 tumors across 33 diverse cancer types identified subtypes, namely wound healing, interferon-gamma, inflammatory, lymphocyte depleted, immunologic quiet and TGF-Beta dominant [86]. These correlated with overall survival, demonstrating cross-talk between cell-intrinsic and cell-extrinsic process. Another TCGA study across 32 cancer types discovered four cycle patterns characterized by “hot”, “cold”, “exhausted”, “inert” or “radical” features with prognostic and predictive capabilities for ICIs [87]. Multiple studies focusing on LUAD arrive at different subtyping schemes, e.g., immunodeficiency vs. immunocompetent; high-risk vs. low-risk, etc. [88,89,90]. But they uniformly elucidate mechanisms showing TIME-cancer cross-talk, e.g., subtype correlation with cell cycle signaling activation, TP53 mutations, and responsiveness to ICIs or anti-angiogenic therapy [88,89,90].

### 3.6. Biomarkers for ICIs

Limitations exist despite the remarkable efficacy of ICIs. A restricted number of patients (12%) achieve durable clinical benefit with monotherapy [91]. There is an increased risk of immune-related drug toxicities, such as the hyperprogression of the tumor with worsened outcomes [92]. The cost of ICI warrants judicious use. These factors demonstrate the necessity for ICI biomarkers.

**Figure 4:**
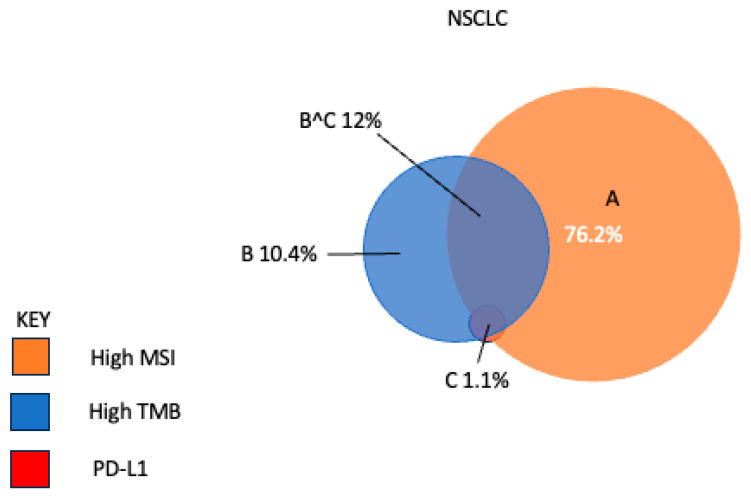
Non-concordance of biomarkers for immune checkpoint inhibitors, MSI, TMB, and PDL-1, imply different underlying immune mechanisms of action in NSCLC. A—high MSI, B—high TMB, C—PD-L1 expression, B^C—overlap between High TMB and PD-L1 expression. Figure reproduced with permission for ESMO Annals of Oncology under the Creative Commons License [104].

The most established biomarkers for ICI responsiveness include PD-1/PD-L1 expression IHC, tumor mutational burden (TMB), microsatellite instability or its equivalent, mismatch repair genes (MMRs) IHC, and tumor-infiltrating lymphocytes [93]. These have distinct yet overlapping predictive properties (Figure 4). Emerging biomarkers include the microbiome [94]. PD-1/PD-L1 IHC is limited by variability in antibodies, scoring methods, cut-offs, platforms, interobserver variability, specimen type and implications for therapy [95,96,97,98,99].

TMB is the total number of mutations per megabase of interrogated genomic sequence in a tumor specimen. TMB is correlated with the likelihood of the production of immunogenic neoantigens [100]. A high proportion of immunogenic neoantigens increases the chances of T cell recognition and cancer cell destruction. Limitations for TMB include variability from methodology, panel size and scoring methods, different implications for tumor type, variable tumor specific cut-offs, etc. [101,102]. Though TMB provides significant prediction benefit in ICIs, a high TMB score does not always equate to immunogenic neoantigen production. Response rates to ICIs in TMB-high tumors is only 45%. TMB assesses the cell-intrinsic property of genomic mutations, while TIME factors including T cell infiltration, the balance between activating and suppressive cytokines and the type of checkpoint exploited by the cancer are not assessed. Host factors outside the domain of TMB that impact immune responsiveness include the MHC class and the TCR landscape. The tumor type impacts TMB interpretation. The response rate in TMB classes shows ICI responsiveness in 5% of TMB-low, 25% of TMB-intermediate, 45% of TMB-high and 65% of TMB-very high.

The genomic factors impact TMB performance. Mutational signatures of mutagenic processes, e.g., UV signatures in chronic sun-damaged melanoma, smoking in lung cancer, aflatoxin B1, viruses and defective MMR (dMMR) frequently produce TMB-high tumors [101]. Cancer-causing viruses, e.g., HPV positive cancers, have higher TMB than their negative counterpart. Mutational signatures, e.g., BRCA1/2, APOBEC deficiency, neoantigen load, TP53 mutations and polymerase ε (POLE) also influence TMB scores.

MMR genes correct DNA replication errors of insertion and deletion arising from base pair mismatch. Clinically relevant genes include MLH1, PMS2, MSH2 and MSH6 [103]. dMMRs cause hypermutation within short tandem repeats in the genome. Screening is performed by IHC or molecular testing by PCR or NGS [104,105].

Major Histocompatibility Complex (MHC) impacts ICI response [106]. Peptide neoantigens are loaded on MHC molecules via the ubiquitin proteosome complex and transported to the cell surface, where they function in antigen recognition with TCR. The maximum heterozygosity of MHCs potentiates host antigen presentation of a higher range of neoantigens. Specific MHC subtypes are innately better at preferentially presenting antigens enriched in tumors. HLA loss of expression and abnormalities of β-microglobulin may result in ICI resistance. MHC functions in the tumor-mediated selection of poorly bound neoantigens in immune evasion. Mutations in MHC may lead to immune evasion. Thus, MHC genotyping is a potential biomarker for immunotherapy.

The TCR repertoire is an important component of ICI responsiveness [107]. High TCR clonality implies that T cell diversity has been correlated with better survival. The absence of T cell response due to impaired or lacking T cell reactivity, or from the active removal of anti-tumor reactive T cells, affects ICI response.

Specific genomic mutations play a role in ICI performance [101]. Genomic alterations, PDL-1 amplification, mutations in serpin genes, CDK12, SMARCA4 and PBRM1, are associated with improved outcomes. Alterations in genes including JAK1/2, STK11 are associated with blunted response. Adverse effects, e.g., hyperprogression, are associated with genetic alterations such as MDM2 amplification and EGFR aberrations.

The complex immune response necessitates a complex biomarker system. A proposed composite biomarker, the immunogram, integrates multiple variables [108]. Components include PDL-1, TMB, dMMR, other checkpoint inhibitors, (TIM-3, LAG-3, VISTA, IDO1), the cell-of-expression, the tumor molecular signatures, HLA genotype, neoantigen immunogenicity, the antigen-presenting capacity of MHC, immune infiltration, cancer microbiome, TCR repertoire, etc. The immunogram would evolve with advances in immunotherapy and immunobiology.

## 4. Computational Biology and AI

Computational biology has established utility in analyzing and integrating high dimensional data from genomics, cancer biology and immunogenomics [109]. Machine learning tools like random forests, support vector machines, boosting, bagging and artificial neural networks have been used extensively to build predictive models for patient prognosis, drug targets identification, linking genetic variants with disease and therapeutic responsiveness. Clustering and dimension reduction algorithms are used in cell-type analysis and tumor characterization. Feature selection algorithms identify the most relevant predictors, while screening out redundancy. Biological modeling beyond machine learning includes mathematical and bioinformatic approaches [110]. Significant biological pathways can be identified from a gene list of mutational data or GEP, e.g., gene set enrichment/over-representation analysis [111]. An integrated network of pathways, drug interactions and clinical data can be constructed by network analysis, producing a personalized profile of patient’s tumor biology [112]. From this profile, inference can be made for prognosis, diagnosis, therapy, monitoring and outcomes [113]. Predictions about resistant mechanisms and compensatory and escape pathways can be deduced to guide combinatorial-targeted therapy.

Nabet et al. developed multi-parametric Bayesian probit models that integrate both tumor-intrinsic and extrinsic features [114]. The pre-treatment composite model (DIREct-Pre) utilized tumor PD-L1 expression, pre-treatment cell-free circulating DNA (ctDNA) and circulating immune cell profiling to accurately predict outcomes in NSCLC patients. Their response classifier, DIREct-On, combined pre-treatment ctDNA, immune profiling, and early on-treatment ctDNA response to predict durable benefit after one cycle of immunotherapy. It could independently predict progression-free survival. These multi-parametric models demonstrated superior performance compared to individual variables.

A multi-variable XGBoost model incorporated 11 meta-analysis-derived predictors consisting of host, tumor and TME factors to predict responders and non-responders based on RECIST criteria. The model was built on a pan-tumor dataset across multiple studies and tumor types, comprising 1008 patients from 12 cohorts. Integrated features included clonal TMB, indel TMB, nonsense-mediated-escape TMB, UV signature, tobacco signature, APOBEC signature, sex, T cell inflamed GEP signature and gene expression values for PD-L1, CD8A and CXCL9. The model performed better that the strongest ICI response predictor, clonal TMB [115].

RF16 is a random forest model that incorporates 16 genomic features with clinical and demographic features to predict response to immunotherapy [116]. This model, derived from 16 different cancer types and 1479 patients, was capable of classifying randomly selected patients with responders showing significantly better overall survival than non-responders. The multi-variate model was superior to individual covariants.

LORIS is an NSCLC-specific logistic regression model that models immunotherapy response from a six-feature-based clinical score [117]. It was developed from over 3000 patients across 18 solid tumor types integrating clinical, pathologic and genomic features. These features include TMB, systemic therapy history, albumin, neutrophil to lymphocyte ratio (NLR), age, cancer type and PD-L1 score. It predicted patient probability for objective response and survival across cancer types, in addition to ICI response. It was capable of identifying responders even in cases with low TMB. It demonstrated superior performance when compared with other, more complex models and individual covariants such as TMB or PD-L1 expression. Its monotonic score and interpretability facilitate translation into clinical decision making and patient stratification.

Deep learning models are capable of significantly improving multimodal integration. DyAM is a dynamic attention-based deep-learning model that incorporates medical imaging, histopathology and genomic data [118]. It makes use of dynamic weighting of features across modalities. It automatically extracts and synthesizes features from disparate modalities, resulting in combinatorial synergy to stratify NSCLC into high- and low-risk groups for immunotherapy. Genomic features derived from a 341-468-gene targeted- NGS assay including mutations in *EGFR*, *ALK*, *ROS1*, *MET*, *ERBB2*, *BRAF*, *STK11*, *ARID1A* and TMB, were utilized in model development. DyAM outputs risks attributed to each modality, the attention the modality receives and an overall score. It achieved superior performance in comparison to established biomarkers and simpler models in predicting PFS and response to PD-L1 blockade in NSCLC patients.

Shen et al. developed COMPASS, a foundational model based on tumor transcriptome data, to predict immunotherapy response [119]. It synthesizes self-supervised learning with a concept bottleneck architecture to derive hierarchical immune representation from bulk RNASeq data. These profiles are mapped to biology-based features including immune cell states, TIME interactions and signaling pathways. In addition to therapy response prediction, it delineates drug resistance mechanisms and generates a patient-specific hypothesis for clinical interpretation and drug trial design. It stratifies patients, identifies mechanisms including cytotoxic T cell activity, interferon-gamma signaling and TGF-Beta pathway activation and predicts an established biomarker such as TMB, PD-L1 score and IHC immune phenotype. Its integration design from pan-cancer transcriptomic pre-training architecture makes it generalizable across cancer types and ICIs while maintaining interpretability. COMPASS was evaluated on 1133 patients from 16 clinical cohorts across seven cancer types, surpassing 22 established models in performance.

## 5. Conclusions

Cancer care has improved with advances in the understanding of cancer biology, immunobiology and therapy. Opportunities exist in fine-tuning diagnosis, drug prediction and prognosis. AI places molecular diagnostics at the cusp of a new multi-omics era, making multidimensional data clinically actionable, and promising a new field for personalized care [111].

## Figures and Tables

**Table 1:** Molecular diagnostics and hallmarks of nonsmall cell lung cancer (NSCLC).

Category	Hallmark/Enabling Characteristic	Examples of Validated/Potential Biomarkers in NSCLC	Examples of Pathways in NSCLC	Validated or Potential Detection Assay
**Core Hallmarks**	Sustaining Proliferative Signaling	*EGFR*, *KRAS*, *BRAF*, *MET*, *CCND1*, *ALK*, *NTRK*, *ROS1*, *RET*, etc.	RAS–RAF–MAPK pathway PI3K–Akt and mTOR pathway	Next Generation Sequencing
	Evading Growth Suppressors	*TP53, RB*	PI3K–Akt and mTOR pathway *BCL2*,	Next Generation Sequencing
	Resisting Cell Death	*TP53*, Noxa, Puma, *MYC*	Autophagic and apoptotic pathways	Gene Expression Profiling, NGS
	Enabling Replicative Immortality	Telomerase, hTERT expression, TERC	TERT regulation, MYC Pathways, Wnt/Beta catenin, mTOR pathway, P53	Telomerase Activity Assay (TRAP) Telomerase Length Assays
	Inducing/Accessing Vasculature	*VEGFR*, *FGF*, *EGF*, *HIF-1 alpha*	PI3K/AKT, RAS/RAF/MEK/ERK pathways	Gene Expression Profiling
	Activating Invasion and Metastasis	COX2, LKB1, WNT, NOTCH, TGF-Beta	WNT, NOTCH, JAK–STAT pathways	Gene Expression Profiling
	Deregulating Cellular Energetics	*HIF-1 alpha*, *MYC*, *TP53*, *SREBP1*	HIF-1 alpha, mTOR, AMPK, MYC, mitochondrial reprogramming	Gene Expression Profiling, ATP Assays, Glucose Uptake Assays, Lactate Production Assays
	Avoiding Immune Destruction	MSI, dMMR, TMB, PDL-1,	PD-L1 pathways, JAK/STAT pathways	Immunohistochemistry, Flow Cytometry, Single Cell Sequencing
**Enabling Characteristics**	Genome Instability and Mutation	Copy number alterations, Amplification, karyotypic instability, *TP53*	DNA repair pathways, e.g., HRD repair pathway P53 pathways, RB	aCGH, SNP Arrays, Karyotyping, HRD Testing
	Tumor-Promoting Inflammation	IL-1, TNF-alpha, macrophage phenotyping	NF-kBeta, STAT3 pathways	Flow Cytometry, IHC, single cell RNA Sequencing
**Emerging Hallmarks**	Unlocking Phenotypic Plasticity	HIF1 alpha, HIF2 alpha, SOX2, Oct4	Cell cycle/DNA damage repair pathways, PRC2 complex, AKT pathways	IHC, Methylation Profiling, NGS, Gene Expression Profiling
	Nonmutational Epigenetic Reprogramming	DNA methylation, miRNA, DNMT1/3A/3B, Histone Deacetylases (HDACs), *EZH2*	DNA methylation, Histone Acetylation, chromatin remodeling complex	Methylation Profiling, Methylation Sequencing
	Polymorphic Microbiomes	Streptococcus, Veillonella, composition of intestinal flora	PI3K/AKT pathway, MAPK pathway	16 s RNA Sequencing, NGS
	Senescent Cells	Senescence-Associated B-galactosidase, DDR markers, Senescence associated secretory phenotype (SASP) markers, e.g., cytokines	DDR, cell cycle inhibitors, MHC class II genes	Gene Expression Profiling, IHC, SASP Profiling, Chromatin Analysis

## Data Availability

No new data was created during this study.
